# i5mC-DCGA: an improved hybrid network framework based on the CBAM attention mechanism for identifying promoter 5mC sites

**DOI:** 10.1186/s12864-024-10154-z

**Published:** 2024-03-05

**Authors:** Jianhua Jia, Rufeng Lei, Lulu Qin, Xin Wei

**Affiliations:** 1 School of Information Engineering, Jingdezhen Ceramic University, 333403 Jingdezhen, China; 2 Business School, Jiangxi Institute of Fashion Technology, 330044 Nanchang, China

**Keywords:** 5mC site, DenseNet, CBAM, BiGRU, Self-attention mechanism

## Abstract

**Background:**

5-Methylcytosine (5mC) plays a very important role in gene stability, transcription, and development. Therefore, accurate identification of the 5mC site is of key importance in genetic and pathological studies. However, traditional experimental methods for identifying 5mC sites are time-consuming and costly, so there is an urgent need to develop computational methods to automatically detect and identify these 5mC sites.

**Results:**

Deep learning methods have shown great potential in the field of 5mC sites, so we developed a deep learning combinatorial model called i5mC-DCGA. The model innovatively uses the Convolutional Block Attention Module (CBAM) to improve the Dense Convolutional Network (DenseNet), which is improved to extract advanced local feature information. Subsequently, we combined a Bidirectional Gated Recurrent Unit (BiGRU) and a Self-Attention mechanism to extract global feature information. Our model can learn feature representations of abstract and complex from simple sequence coding, while having the ability to solve the sample imbalance problem in benchmark datasets. The experimental results show that the i5mC-DCGA model achieves 97.02%, 96.52%, 96.58% and 85.58% in sensitivity (Sn), specificity (Sp), accuracy (Acc) and matthews correlation coefficient (MCC), respectively.

**Conclusions:**

The i5mC-DCGA model outperforms other existing prediction tools in predicting 5mC sites, and it is currently the most representative promoter 5mC site prediction tool. The benchmark dataset and source code for the i5mC-DCGA model can be found in https://github.com/leirufeng/i5mC-DCGA.

## Introduction

Epigenetic inheritance [1] is a heritable change in the function of a gene in the absence of a change in the DNA sequence of the gene, and eventually leads to a change in phenotype [2]. In epigenetics, DNA methylation [3] is an important modification that plays a profound role in gene stability, transcription, and development. DNA methylation is under the catalyzed action of DNA methyltransferases (DNMTs) [4] to transfer of a methyl group to the fifth carbon of cytosine (C) of the CpG dinucleotide of the genome [5, 6]. There are several types of DNA methylation modification sites, including Adenine N-6 methylation (6 mA), Cytosine N-4 methylation (4mC), Guanine N-7 methylation (m7G) and Cytosine C-5 methylation (5mC) [7, 8]. Many of them occur on the CpG island in the promoter region of the gene and are catalyzed by different DNA methylation enzymes. Methylation modification of the 5mC sites influences chromatin structure and can also regulate gene accessibility and expression levels. Methylated sites interfere with the binding of transcription factors to DNA, thereby affecting the transcriptional activity of genes. In addition to this, methylation modification of the 5mC site plays a crucial regulatory role during cell development and differentiation. During this process, specific genes undergo demethylation, which can deregulate the repressive effects of genes and thus promote cell differentiation. Meanwhile, methylation modifications of the 5mC site are influenced by environmental factors such as external stimuli and stress, thereby regulating gene expression to adapt to environmental demands. 5mC is the earliest type of methylation found in eukaryotes, it represses the transcriptional activity of genes, and it is strongly associated with the production of diseases such as genetic disorders, cancers, and tumors [9]. Therefore, accurate identification of the 5mC site in the promoter region is important in genetic and pathological studies.

In the past, researchers usually used traditional experimental methods [10] to identify 5mC sites, which are time-consuming and expensive. Therefore, there is an urgent need to develop computational methods to automatically detect and identify these 5mC sites, providing biologists with more efficient and convenient tools. In recent years, an increasing number of models based on machine learning constructs have been used to identify 5mC sites, such as Stacking Ensemble [11], XGBoost [12] and SVM [13, 14]. These models have greatly facilitated the study of the 5mC site, allowing researchers to probe this methylation type in greater depth. However, these machine learning models require complex feature encoding to improve their performance. Developing complex feature encodings is a time-consuming and labor-intensive task. Therefore, there is an urgent need to develop a series of 5mC site predictors that use simple feature encoding methods to improve prediction accuracy and reduce the complexity of feature encoding.

In recent years, deep learning has been widely used in the field of 5mC sites and has achieved remarkable results. Deep learning models [15] can automatically learn feature representations of abstract and complex from simple sequence coding [16], and it has excellent predictive and generalization performance. In 2020, Zhang et al. [17] created a dataset containing DNA promoter 5mC sites. The ratio of positive and negative samples in this dataset is 1:11. To deal with this unbalanced dataset, they developed a model called iPromoter-5mC. This model uses manual segmentation to divide the negative samples into 11 parts and uses 11 fully connected neural networks for voting integration to arrive at the final classification result. Based on the idea of iPromoter-5mC model, Cheng et al. [18] developed a model named BiLSTM-5mC. In 2023, Jia et al. [19] developed a model named DGA-5mC. The model can automatically process unbalanced 5mC site data without manual processing. But they also used the voting integration method to arrive at the final classification. In the meantime, in the field of the 5mC site of RNA, Hassan et al. [20] developed a model named Deepm5C. Shi et al. [21] developed a model named R5hmCFDV. They both used several deep learning methods to predict 5mC sites through an integrated strategy of Stacking and Voting [22]. All the above prediction models have achieved remarkable results in the field of 5mC sites and have enriched the study of 5mC sites. We found that using better deep learning networks improves prediction accuracy, indicating that there is still much space for innovation in the combination of neural networks. All the models mentioned above use an ensemble learning to improve model performance. However, this also leads to an increase of network parameters and consumption of computational resources, and they may not be feasible when dealing with large benchmark datasets.

In the field of bioinformatics, Convolutional Neural Network (CNN) [23, 24] are often used to extract local feature information from the original sequence. However, CNN have problems such as gradient vanishing and network degradation. Researchers prefer to use Residual Neural Network (ResNet) [25] and Dense Convolutional Network (DenseNet) [24]. ResNet uses a Residual block structure to prevent the problems of gradient vanishing and network degradation by short-circuiting the connections. Based on ResNet, DenseNet proposes a Dense block structure, which uses the dense connection mechanism to reuse the feature information of all previous layers. Therefore, DenseNet is more excellent in terms of effect compared to ResNet. Furthermore, Recurrent Neural Network (RNN) [26] are often used in extracting global feature information from the original sequence. However, when dealing with long sequence features, RNN have the problem of gradient vanishing or gradient explosion. To overcome this problem, researchers have designed Long Short-Term Memory Network (LSTM) [27] and Gated Recurrent Unit (GRU) [28, 29], which ameliorate the drawbacks of RNN to some extent. In addition, more and more researchers have started to explore and apply attention mechanisms. Attention mechanisms can be flexibly applied in various types of neural networks, including Convolutional Block Attention Module (CBAM) [30] and Self-Attention mechanisms [31]. These attention mechanisms automatically weight the output feature maps to highlight useful feature information, thus improving the performance and robustness of the model.

Xiao et al. [32] further extended Zhang et al.‘s promoter 5mC site dataset to several million. In addition, they developed a Stacking integration model based on a machine learning approach called m5C-HPromoter. This model utilizes the SMOTE method [33] to transform unbalanced data into balanced data and has achieved good results in experiments. In this study, based on Xiao et al. constructed benchmark dataset, we develop a deep learning combinatorial prediction model called i5mC-DCGA. The model uses an improved DenseNet to extract advanced local feature information. Subsequently, we combine a Bidirectional Gated Recurrent Unit (BiGRU) and a Self-Attention mechanism to extract global feature information. Our model can extract richer feature information and far exceeds existing prediction models in terms of performance. Therefore, the i5mC-DCGA prediction model is currently the best choice for predicting promoter 5mC sites.

## Results and discussion

### Construction of model

**Fig. 1:**
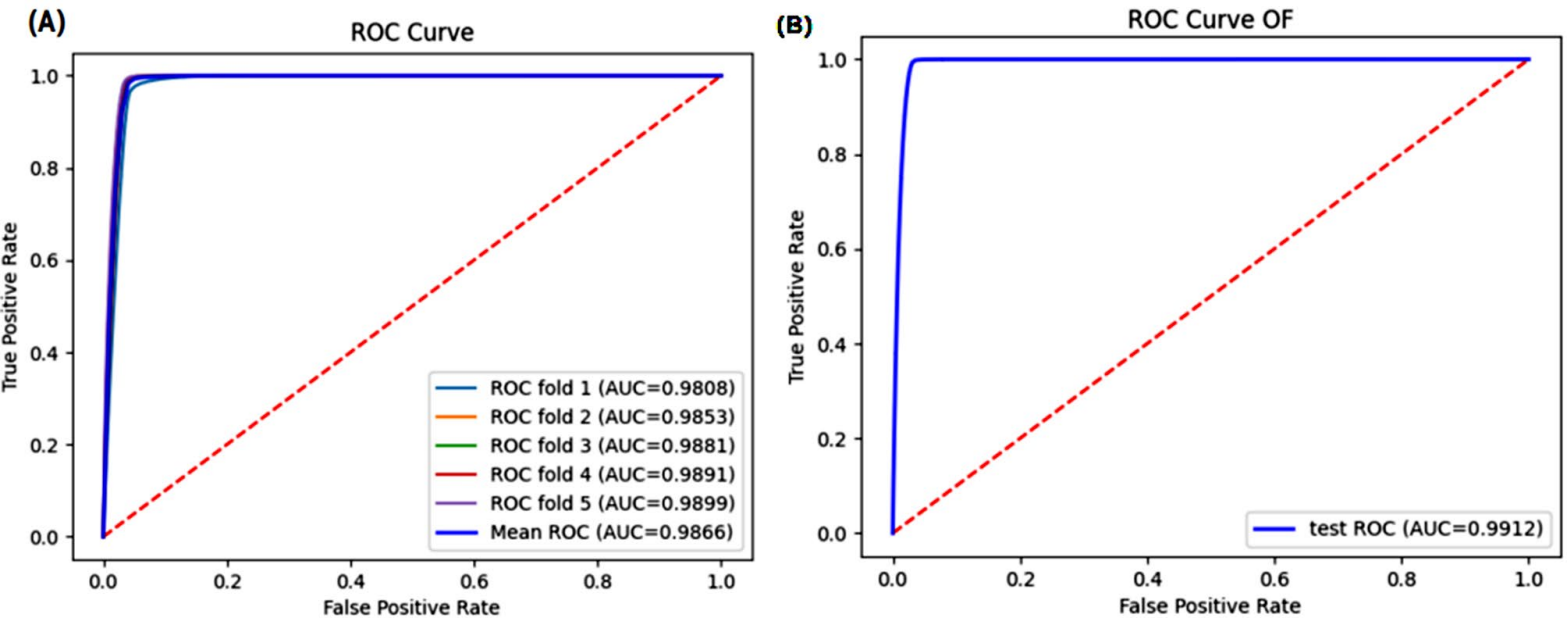
(**A**) ROC curves of 5-fold cross validation, (**B**) ROC curves of independent test

In this study, we developed a novel deep learning model called i5mC-DCGA. The model has the ability for automatic extraction of high-level features and network weight propagation, as well as the capability to automatically deal with sample imbalances present in the baseline dataset. First, we coded the promoter 5mC site sequence using One-hot and NCP coding methods, and then merged the two feature codes. The merged feature codes are fed into an improved DenseNet to extract advanced local feature information. Next, we use BiGRU and Self-Attention to extract global feature information. Finally, we input the global feature information into the fully connected neural network to derive the predicted probabilities and calculate the evaluation metrics. We obtained average experimental results using 5-fold cross-validation with 97.02% for Sn,96.52%for Sp,96.58%for Acc, and 85.58% for MCC. And in independent tests, our experimental results were 99.50% for Sn, 96.79% for Sp, 97.11% for Acc, and 87.79% for MCC. Figure 1 shows the ROC curves for 5-fold cross-validation and independent testing. The results show that our proposed i5mC-DCGA model has good robustness.

### Compare the number of dense blocks

**Fig. 2:**
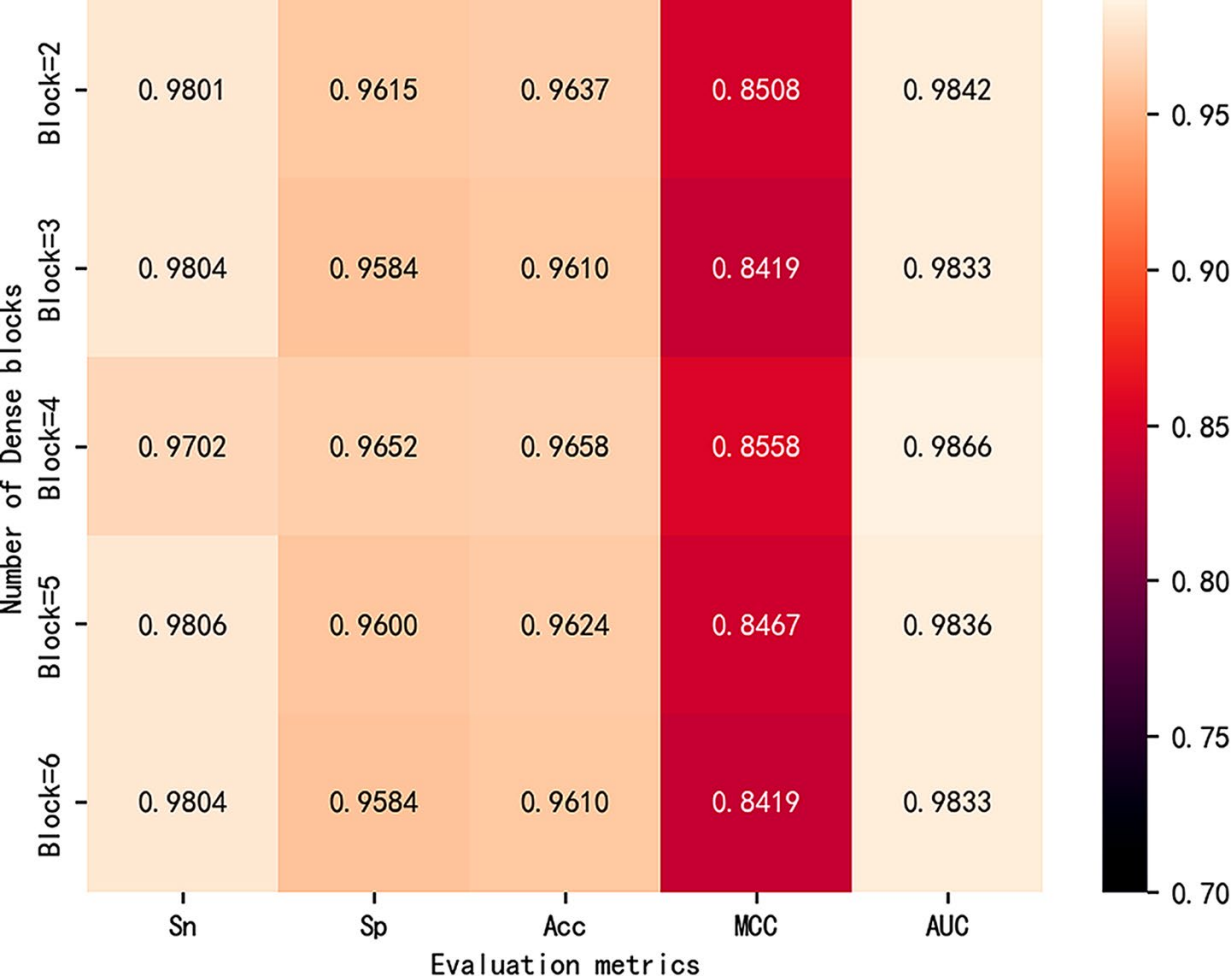
Evaluation metrics for different number of dense blocks

In this study, the improved DenseNet is the main advanced local feature extraction network. However, in the improved DenseNet, the number of Dense blocks is an important factor in the model performance. If the number of Dense blocks is too much or too little, it can lead to overfitting or underfitting problems in the model. Therefore, choosing the number of Dense blocks appropriately is crucial for obtaining good model performance. In the i5mC-DCGA model, keeping the other neural network architectures and parameters unchanged, we compare the performance of the models by adjusting the number of Dense blocks. According to the experimental results in Fig. 2, when the number of Dense blocks is set to 4, the model reaches the best level in the evaluation metrics of Sp, Acc, MCC and AUC. It is also observed that when the number of Dense blocks is increased, all the evaluation metrics of the model show a decreasing trend, which indicates that there is overfitting of the model. Therefore, we choose four Dense blocks to construct the improved DenseNet network.

### Comparison of different coding methods

**Fig. 3:**
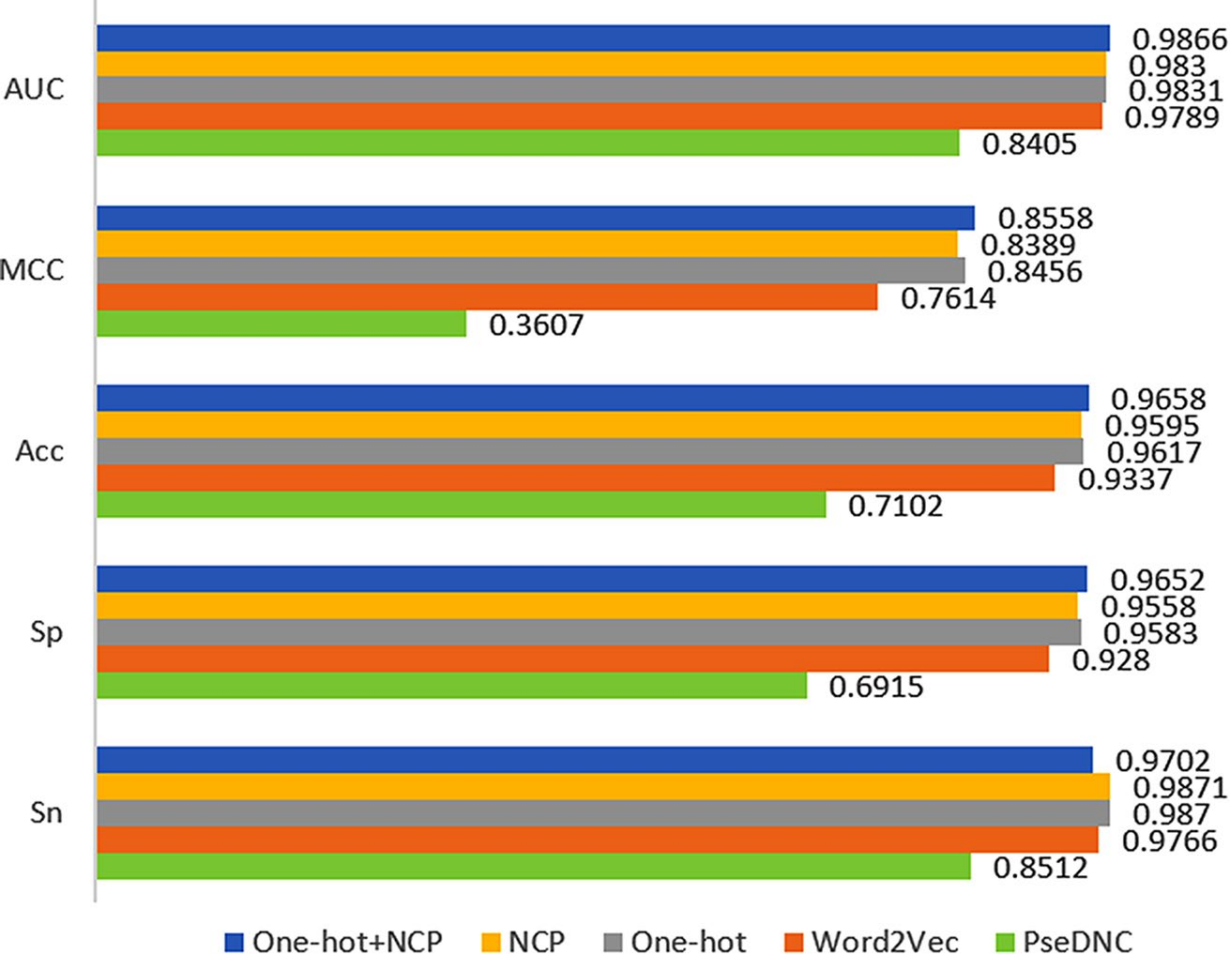
Comparative results of different coding schemes

We have been committed to developing models that use only simple feature coding. In this study, our network framework needs to accept feature codes as input data in the form of a two-dimensional matrix. However, current mainstream feature coding methods typically encode DNA sequences in the form of individual vectors, which does not meet the input format requirements of our network framework. Therefore, we proposed three simple feature matrix coding methods and one complex feature matrix coding method, including One-hot coding, NCP coding, One-hot + NCP coding and Word2Vec coding [34]. Simple feature codes have only two numbers 0 and 1, they are very simple and efficient. Word2Vec coding is a word vector coding method based on contextual semantic information trained on the network. To further validate our network framework, we also conducted comparison experiments with PseDNC coding [35], which provides information about specific patterns and features in the sequence. We put these five coding methods into the i5mC-DCGA modelling framework. According to the experimental results in Fig. 3, the complex Word2Vec coding is less effective compared to other matrix coding methods. The experimental results of One-hot coding were slightly better than those of NCP coding. The MCC and Acc of One-hot coding is 0.8456 and 0.9617, respectively, while the MCC and Acc of One-hot + NCP coding is 0.8558 and 0.9658, respectively. It is evident that there is a significant difference between the two coding on the MCC metrics. All things considered, One-hot + NCP coding performs slightly better than One-hot coding, even though it consumes slightly more computational resources. Therefore, we believe that it is worthwhile to invest such resources. However, One-hot + NCP coding performs best in terms of classification performance, suggesting that under big data-driven conditions, the model can automatically learn meaningful feature information without the need to manually construct complex feature coding. Furthermore, the drawback of Word2Vec coding is that the model parameters become large, leading to increased resource consumption and training time. According to the experimental results, it is found that PseDNC coding performs relatively poorly in deep learning networks and has a weak effect compared to other coding methods. This may indicate that the complex PseDNC coding is not effective in allowing deep learning networks to automatically extract valid feature information from sequences when dealing with large datasets. Therefore, we identified One-hot + NCP coding as the preferred solution for feature engineering.

### Comparison of different model frameworks

We performed ablation experiments on the network framework of the i5mC-DCGA model. We also compare the performance of three network structures, ResNet, primitive DenseNet and improved DenseNet. At the same time, we also compared our model with two machine learning classifiers. The experimental results are shown in Table 1, where the improved DenseNet network significantly outperforms ResNet and the primitive DenseNet in all evaluation metrics, which indicates that the improved DenseNet network can extract more advanced feature information. Furthermore, combining the improved DenseNet network with the BiGRU network demonstrates excellent experimental results. This shows that fusing local and global features can significantly improve performance. Meanwhile, we find that the introduction of the Self-Attention mechanism can improve the robustness of the model. Compared to machine learning models, our model has a significant performance advantage, especially in predicting positive samples. This indicates that deep learning models can predict positive samples more accurately, thus improving the overall level of classification performance. According to the experimental results, the network framework of “Imp DenseNet + BiGRU + Self-Attention” performs the best. Therefore, we finally chose this network framework as the optimal model.

**Table 1:** Comparison of different network architecture models

Model frameworks	SN	SP	Acc	MCC	AUC
AdaBoost	0.6192	0.9655	0.9250	0.6183	0.7923
Random Forest	0.7537	0.9677	0.9427	0.7220	0.8607
ResNet	0.9702	0.9151	0.9215	0.7274	0.9770
Pri DenseNet	0.9754	0.9148	0.9219	0.7301	0.9774
Imp DenseNet	0.9843	0.9276	0.9342	0.7643	0.9795
Imp DenseNet + BiGRU	0.9719	0.9634	0.9644	0.8513	0.9849
GRU + Self-Attention	0.9836	0.9560	0.9592	0.8370	0.9806
Imp DenseNet + Self-Attention	0.9874	0.8976	0.9081	0.7035	0.9768
Imp DenseNet + BiGRU + Self-Attention	0.9806	0.9603	0.9626	0.8475	0.9837
Imp DenseNet + BiGRU + Self-Attention	0.9702	**0.9652**	**0.9658**	**0.8558**	**0.9866**

### Comparison of i5mC-DCGA with existing predictors

In recent years, many excellent computational methods have been emerging in the field of 5mC sites. To evaluate the performance of the i5mC-DCGA model, in this study we compare it with existing models on a benchmark dataset. We used 5-fold cross-validation and independent testing and displayed the results in Table 2. The results showed that the i5mC-DCGA model was significantly better than the existing models in four important evaluation metrics (Sn, Sp, Acc and MCC). This shows that the i5mC-DCGA model has more stable and superior performance. It can predict 5mC sites more accurately and reliably. Meanwhile, we tried the latest 5mC site prediction model DGA-5mC on the benchmark dataset of this study. However, we found that the parameter number of DGA-5mC model is as high as 23 million, which requires a lot of resources and time to train on a large-scale dataset, while our model has only 3.3 million parameters. Therefore, due to these limitations, we decided to abandon further comparisons and studies using the DGA-5mC model. As a result, the i5mC-DCGA prediction model not only excels in performance, but also has higher efficiency and resource utilization.

**Table 2:** Compared with other methods on the same benchmark datasets

Predictor model	SN	SP	Acc	MCC	AUC	F1_score	PR-AUC
iPromoter-5mC(5-fold)	0.8578	0.8641	0.8629	0.6340	------	------	------
m5C-Hpromoter(5-fold)	0.8871	0.9048	0.9023	0.6831	------	0.7485	------
i5mC-DCGA(5-fold)	**0.9702**	**0.9652**	**0.9658**	**0.8558**	**0.9866**	**0.8716**	**0.8611**
i5mC-DCGA(test)	**0.9950**	**0.9679**	**0.9711**	**0.8798**	**0.9912**	**0.8857**	**0.8822**

At the same time, Xia et al. also performed independent test experiments across cellular tissues. They utilized data from small cell lung cancer and non-small cell lung cancer for model training, while data from hepatocellular carcinoma served as an independent test dataset. We compared the i5mC-DCGA model with existing models on this independent test dataset. Table 3 shows the results of the independent test. The range of improvement of our i5mC-DCGA model in Sn is 8.19–10.20% and in Sp is 3.10–4.53%. In addition, the range of improvement of our model in ACC and MCC is 3.76–5.26% and 14.73–19.26% respectively. On independent test datasets, our i5mC-DCGA model showed excellent performance, and thus it is expected to be the most representative tool for predicting the promoter 5mC site.

**Table 3:** Compared with other models on the same independent datasets

Predictor model	SN	SP	Acc	MCC	AUC
iPromoter-5mC	0.8922	0.9147	0.9120	0.6781	------
m5C-HPromoter	0.9123	0.9290	0.9270	0.7234	------
i5mC-DCGA	**0.9942**	**0.9600**	**0.9646**	**0.8707**	**0.9841**

### Comparison of i5mC-DCGA with existing predictors in other datasets

**Fig. 4:**
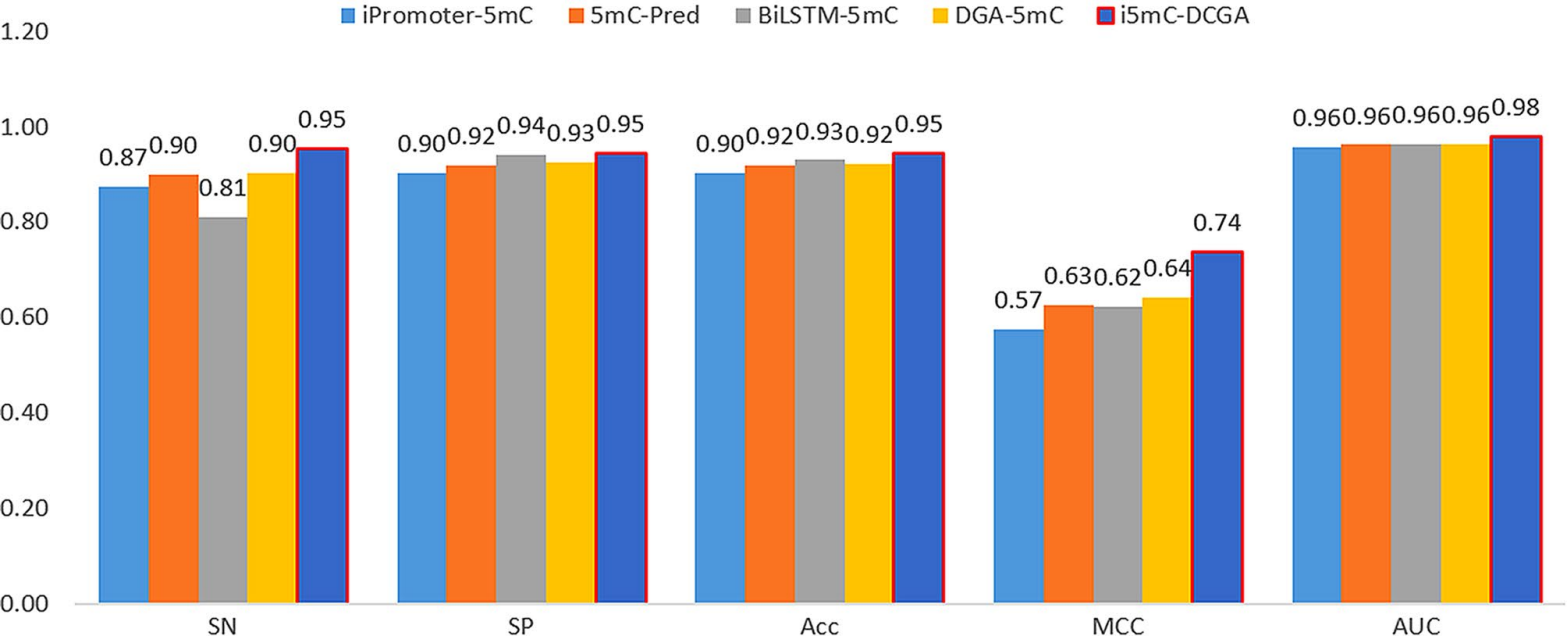
The Zhang et al. dataset is compared with other models

In addition, to further validate the performance of our model, we tested the i5mC-DCGA model again on the benchmark dataset of Zhang et al. This benchmark dataset contains 69,750 positive samples and 823,576 negative samples, and they randomly divided the positive and negative samples into a training set and an independent test set in the ratio of 8:2. The experimental results are shown in Fig. 4, and our model also far outperforms the other four predictors in various metrics. Among them, the most important evaluation metric MCC was improved in the range of 9.57-16.31%. This indicates that the i5mC-DCGA model exhibits good robustness and generalization performance in the task of predicting other 5mC sites, making it the cutting-edge 5mC site prediction model.

## Conclusions

We developed a new promoter 5mC site prediction model called i5mC-DCGA. The model is based on an improved DenseNet, BiGRU, and Self-Attention mechanism combination implementation. The experimental results showed that the i5mC-DCGA model exhibited significant experimental results in predicting the 5mC site. Compared with other existing models, the i5mC-DCGA model demonstrated excellent performance in all four-evaluation metrics (Sn, Sp, Acc, and MCC), which proved that the i5mC-DCGA model possessed excellent generalization ability and performance. We are committed to combining deep learning methods with 5mC methylation studies to enrich the research progress in this field. The i5mC-DCGA model not only has the application potential in the field of promoter 5mC methylation, but also can be applied to the prediction tasks in other DNA sequence fields. This will provide researchers more convenient tools to facilitate the research development in related fields.

Although, our proposed i5mC-DCGA model performs very well, there are still some issues to be addressed. Since our prediction task is based on a large-scale benchmark dataset using a very complex network structure, the model is not suitable for training on small sample datasets. In the meantime, we regret not having constructed an online prediction website. However, we have made the source code publicly available for use by interested researchers. With further research on promoter 5mC methylation, we expect more advanced deep learning methods to appear, bringing more possibilities for promoter 5mC site prediction.

## Materials and methods

### Benchmark dataset

The construction of the benchmark dataset is important for developing the model. In this study, we used the Xiao et al. created a benchmark dataset that incorporates promoter 5mC site data from the Cancer Cell Line Encyclopedia (CCLE) database [36]. The dataset includes a variety of cancer types, such as human small cell lung cancer, human non-small cell lung cancer, and human liver cancer. After experimental validation, they demonstrated that the appropriate length of the promoter 5mC site sequence is 41 bp. In addition, to ensure the uniqueness of each sample, they used CD-HIT [37] to remove promoter 5mC site sequences with more than 80% similarity. The benchmark dataset consists of 386,773 real 5mC site samples and 2,923,874 spurious 5mC site samples. The ratio of positive and negative samples is approximately 1:7.6, showing an unbalanced state. The details of the benchmark dataset are shown in Table 4. In order to ensure an unbiased evaluation of model performance, we randomly divided the positive and negative samples into a training set and an independent test set at a ratio of 8:2. We used stratified random sampling method by randomly dividing 20% of the positive sample as the positive sample of the independent test set and also randomly dividing 20% of the negative sample as the negative sample of the independent test set. Such an approach ensures a representative and fair sample.

**Table 4:** The details of the benchmark dataset

Original dataset	Positive	Negative
Human small cell lung cancer	69,750	823,576
Human non-small cell lung cancer	170,484	1,164,674
Human hepatocellular carcinoma	146,539	935,624
Total	386,773	2,923,874

### Feature coding schemes

#### One-hot coding

One-hot coding [38] is the most used feature coding methods in the field of bioinformatics, which possesses the advantages of simplicity and efficiency. One-hot coding can code four deoxyribonucleotides into binary vectors and the four vectors are independent and non-repeating. One-hot coding is the most primitive expression of a DNA sequence and is very easy to implement. It codes each nucleotide in the order “ACGT”, where the position of the nucleotide is coded as 1 and the other positions are coded as 0. For example, nucleotide A was coded as (1, 0, 0, 0), nucleotide C was coded as (0, 1, 0, 0), nucleotide G was coded as (0, 0, 1, 0), and nucleotide T was coded as (0, 0, 0, 1). One-hot coding of each promoter 5mC site sequence generated a feature matrix of size 4 × 41.

#### NCP coding

NCP coding [39] is a coding approach based on the chemical properties of the nucleotides, which makes use of the different chemical molecular structures of the four nucleotides (ring structure, functional groups, and hydrogen bond strengths) to rationalize the coding [40]. The specific chemical properties and corresponding codes between the nucleotides are shown in Table 5.

**Table 5:** Nucleotide chemical property

Chemical property	Category	Nucleotide	corresponding code
Ring structure	Purine	A, G	1
	Pyrimidine	C, T	0
Functional group	Amino	A, C	1
	Keto	G, T	0
Hydrogen bonding	Strong	C, G	1
	Weak	A, T	0

NCP codes each nucleotide as: A (1, 1, 1), C (0, 1, 0), G (1, 0, 0), T (0, 0, 1). NCP coded each promoter 5mC site sequence to generate a feature matrix of size 3 × 41.

### Model construction

**Fig. 5:**
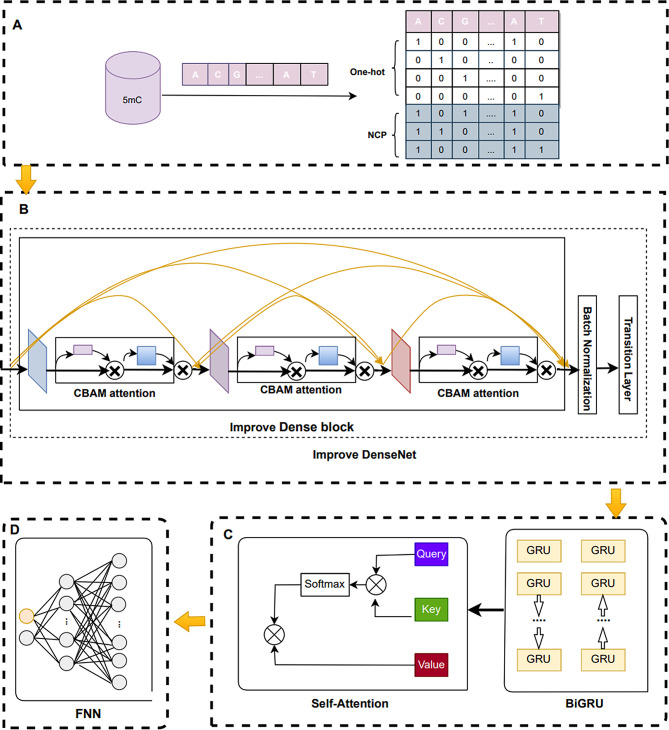
An overview of i5mC-DCGA model. (**A**) Feature coding. (**B**) Improved DenseNet network. (**C**) BiGRU and Self-Attention mechanisms. (**D**) Classification

In this study, we develop a new deep learning model called i5mC-DCGA. The model is capable of automatically learning feature information of abstract and complex from simple sequence codings and can deal with the sample imbalance problem in the benchmark dataset. The general framework of the i5mC-DCGA model is shown in Fig. 5. It is divided into four parts: (A) Feature coding. We code the promoter 5mC sequence using One-hot and NCP respectively to generate two feature coding matrices. These two feature matrices are combined into a feature matrix of size 7 × 41. (B) Improved DenseNet network. We directly input the 7 × 41 feature matrix into the improved DenseNet network to extract more advanced local feature information. This improved DenseNet network is using the CBAM attention mechanism to evaluate the output feature maps of each convolutional layer in a dense block as a means of extracting more advanced local feature information. (C) BiGRU and Self-Attention mechanisms. We use BiGRU and Self-Attention mechanisms to extract global feature information. In this way, both local and global features are extracted in our model. The final output feature information is a combination of these two features, which makes the feature information richer. (D) Classification. The final Self-Attention mechanism outputs feature information fed into the fully connected layer and the SoftMax activation function is used to derive the predicted probability.

#### Improve DenseNet

The structure of the improved DenseNet network has been changed from the primitive DenseNet. The primitive DenseNet contains one Convolutional layer, multiple layers of Dense blocks and Transition layers. Instead, we modified the primitive DenseNet by removing a convolutional layer so that feature coding can be directly input to the Dense block layer. In addition, we introduce the CBAM attention mechanism for evaluating the feature maps of each convolutional layer in a Dense block. The CBAM attention mechanism can evaluate the feature maps in terms of channel and spatial dimensions. In addition, Batch Normalization [41] was added between Dense blocks and Transition layers. With these improvements, our DenseNet can extract more advanced feature information and improve the robustness of the model.

#### Improve dense block

**Fig. 6:**
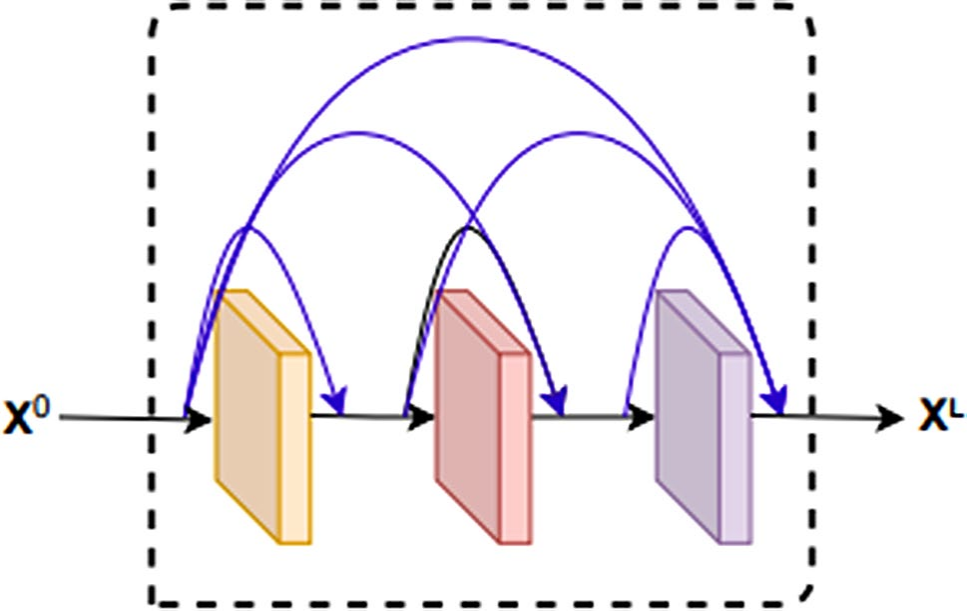
The structure of a dense block

The DenseNet network is based on the residue structure of ResNet, which uses a densely connected structure. The densely connected structure achieves feature reuse by concatenating the outputs of all previous layers by channel dimension as inputs to the next convolutional layer. The Dense block structure improves the efficiency of the model and enhances the expressive power of the model. The Dense block structure is shown in Fig. 6.

**Fig. 7:**
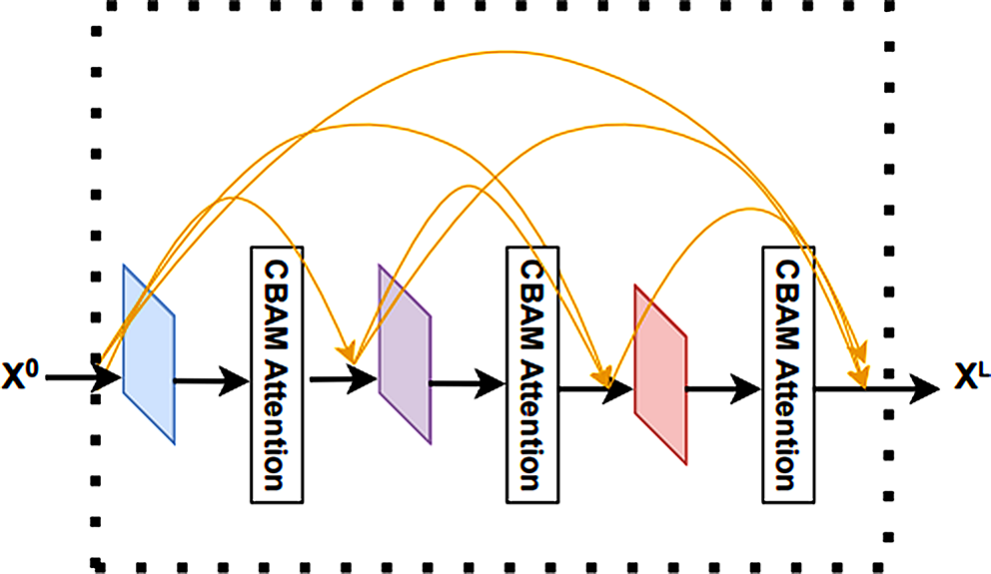
The structure of a improve dense block

We have improved the DenseNet network structure, mainly by introducing the CBAM attention mechanism in the Dense block. The CBAM attention mechanism evaluates the feature maps output from each convolutional layer in the Dense block, which includes the channel dimension and the spatial dimension. It suppresses useless feature information in the feature map, which improves the performance of the model. By introducing the CBAM attention mechanism, our improved DenseNet network can more accurately capture important features and better adapt to the 5mC site prediction task. The improved Dense block structure is shown in Fig. 7.

In a Dense block, the inputs to the $$ {X}_{l}$$ layer are related to the outputs of all previous layers, and $$ {X}_{l}$$ is represented as follows:

1 $$ \begin{array}{c}{X}_{l}={H}_{l} \left(\left[{X}_{0}{,X}_{1}{,X}_{2},\dots,{X}_{l-1}\right]\right)\end{array}$$

where [] denotes the concatenation operation. ${H}_{l}(\cdot )$ is the nonlinear transformation function, which consists of Batch Normalization, ReLU activation function and Convolution (3 × 3).

#### Transition layer

In DenseNet, to solve the problem of large number of feature map channels and parameter explosion, we use Transition layer to optimize this problem. The Transition layer consists of a 1 × 1 convolution and a 2 × 2 average pooling. With 1 × 1 convolution, we can reduce the number of channels in the feature map and decrease the complexity of the model. Meanwhile, the 2 × 2 average pooling reduces the size of the feature map, further reducing the number of parameters and computation. By introducing the Transition layer, the DenseNet network retains rich feature information while improving performance and training efficiency.

We introduced a Batch Normalization layer in the Transition layer. The feature map is processed by the Batch Normalization layer, which can effectively alleviate the parameter explosion problem. In addition, the Batch Normalization can accelerate model training and improve the generalization ability of the model. By applying Batch Normalization in the Transition layer, we can further improve the DenseNet model, making it more stable, with better performance and stronger generalization capabilities.

#### CBAM attention mechanism

CBAM attention mechanism is a simple and effective module that can be easily integrated into any CNN architecture. It consists of two modules, Channel attention mechanism and Spatial attention mechanism, so it can focus on important features and suppress unnecessary features in both channel and spatial dimensions. Firstly, the original sequence features will be passed through the Channel attention module and the channel-weighted feature map will be fed into the Spatial attention module. Finally, the final weighted feature map output by the Spatial attention module. The CBAM attention module can enhance the model’s representation of the original sequence features.

#### Channel attention module

**Fig. 8:**
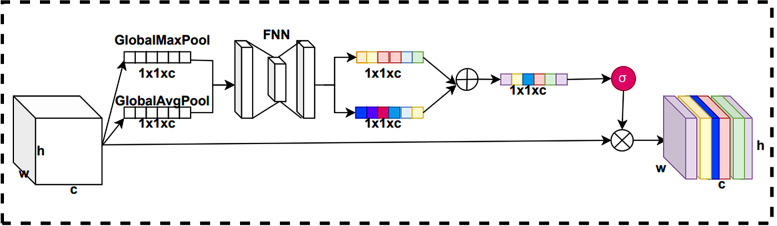
Channel attention module of CBAM.

The importance of different channel information of the feature map varies. The Channel attention module is mainly used to process the different channel information in the input feature map. In traditional convolutional neural networks, the weights of each channel are fixed, and adaptive learning based on the importance of features is not possible. In the Channel attention module, the weights of each channel can be adaptively learned based on the importance of the features to improve the performance of the model. This means that the model can be more flexible to focus on important channel features. By introducing the Channel attention module, the model can better utilize the information of different channels, thus improving the representation of features and the performance of the model. The structure of the Channel attention module of CBAM is shown in Fig. 8.

The Channel attention module compresses the original feature map using global maximum pooling and global average pooling to obtain feature vectors of two channel dimension sizes. These two feature vectors are trained by a shared fully connected neural network. After training, the two feature vectors are summed with their corresponding elements. The summed vectors are normalized by a sigmoid activation function to generate the weight of each channel. Finally, the normalized weights are multiplied with the input feature map.

#### Spatial attention module

**Fig. 9:**
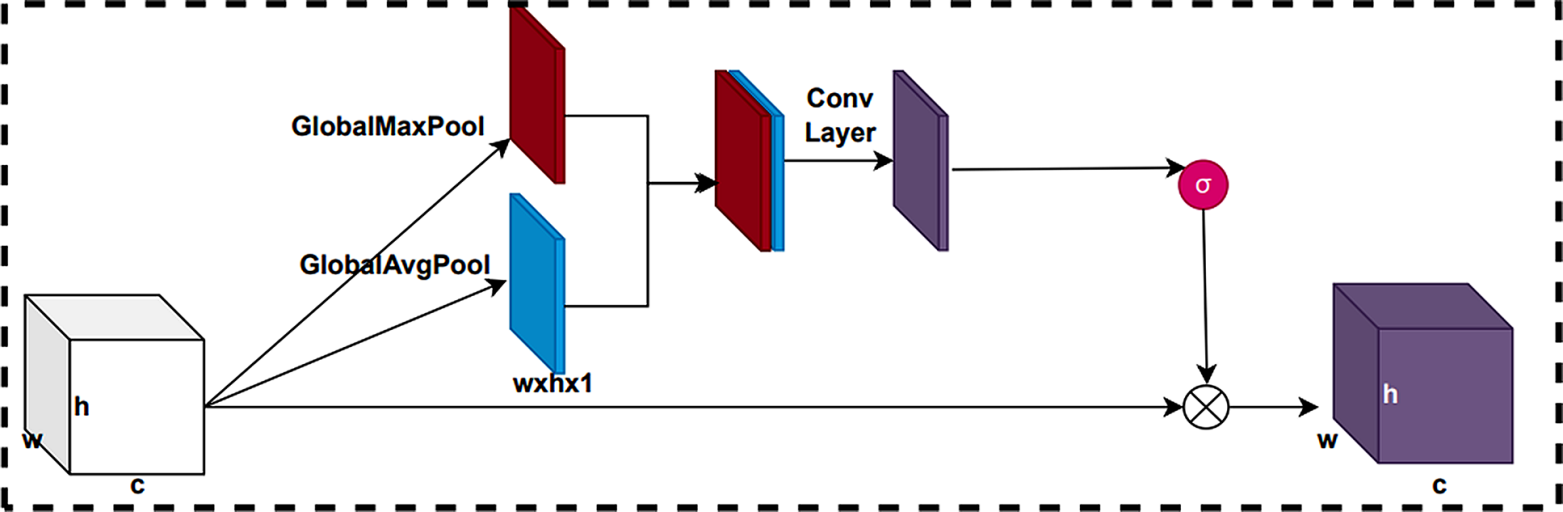
Spatial attention module of CBAM.

There are differences in the importance degree of different locations of the feature map. To deal with this discrepancy, we employ a Spatial attention module. The Spatial attention module can adaptively learn the weights of each spatial location based on the importance of the features to improve the model performance. The structure of the Spatial attention module of CBAM is shown in Fig. 9.

This module takes the feature map output from the Channel attention module as the input feature map. First, we can perform channel dimension compression by using global maximum pooling and global average pooling operations on the input feature maps. This results in two feature matrices of size $W\times H\times 1$. Then, we will concatenate these two feature matrices in the channel dimension and train them by convolutional neural network in return to obtain a training feature matrix. The generates spatial attention weights by applying sigmoid activation function to this training feature matrix for normalization operation. Finally, this spatial attention weight is multiplied element-by-element with the input feature matrix to obtain the final feature map.

#### BiGRU

**Fig. 10:**
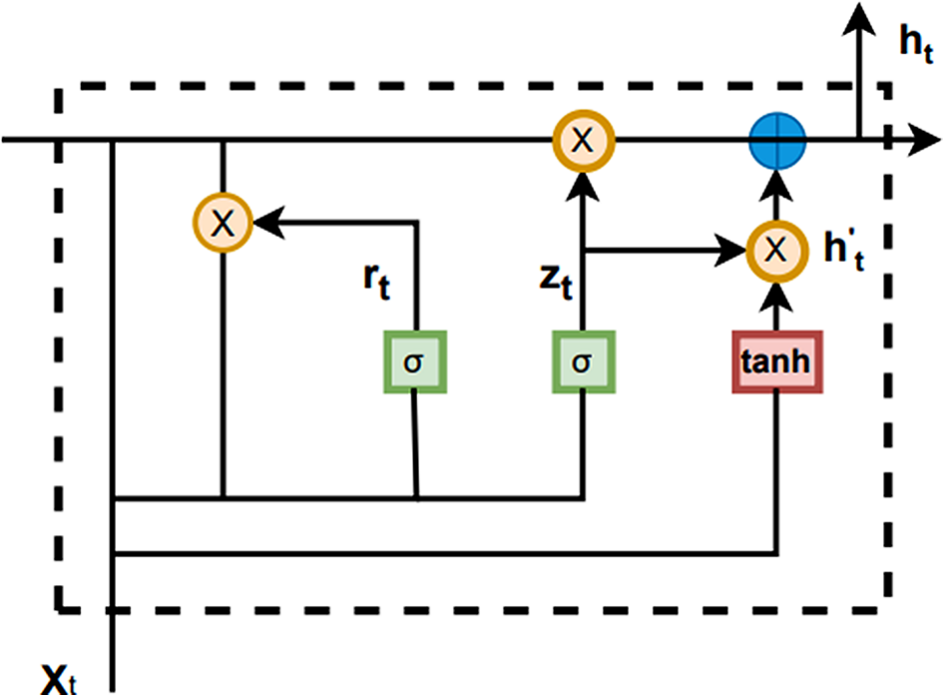
Internal structure of GRU.

Traditional RNN can capture global information about the original sequence features, but it is prone to the problems of gradient vanishing and gradient explosion. To solve this problem, LSTM introduces a special gating mechanism that effectively captures the long-term dependencies in the original sequence features. GRU simplifies the gating mechanism of LSTM to improve training efficiency. The GRU structure is shown in Fig. 10. BiGRU by processing the input sequence in the forward and backward direction, it can solve the problem of gradient vanishing and gradient explosion more effectively, thus improving the accuracy and stability of the model.

The GRU model uses the last hidden state $$ {h}_{t-1}$$ and the input $$ {x}_{t}$$ of the current node to compute the reset gate (denoted as $$ {r}_{t}$$) and the update gate (denoted as $$ {Z}_{t}$$). These two gating signals are compressed into weights by a sigmoid activation function for controlling the information flow. First, we can use the previous hidden state $$ {h}_{t-1}$$, $$ {r}_{t}$$ and $$ {x}_{t}$$ computations to obtain the temporary hidden state $$ {h}_{t}^{{\prime }}$$. Finally, a weighted summation of $$ {h}_{t}^{{\prime }}$$ and $$ {h}_{t-1}$$ under the action of $$ {Z}_{t}$$ yields the final hidden state $$ {h}_{t}$$. If the gating signal of update gate is close to 1, it represents that more information of the current node is memorized. On the contrary, if it is close to 0, it means that more information of the current node is forgotten. The exact formula is shown below:

2 $$\eqalign{& {r_t} = \sigma ({W_z}{x_t} + {U_z}{h_{(t - 1)}}) \cr & {z_t} = \sigma ({W_z}{x_t} + {U_z}{h_{(t - 1)}}) \cr & {{h{^{\prime}}}_t} = tanh(W{x_t} + U({r_t} \odot {h_{(t - 1)}})) \cr & {h_t} = (1 - {Z_t}) \odot {h_{(t - 1)}} + {Z_t} \odot {{h{^{\prime}}}_t} \cr} $$

where all $$ W$$ and $$ U$$ correspond to the weight matrices at the positions, $$ \sigma $$is the sigmoid activation function, and $$ tanh$$ is the hyperbolic tangent activation function.

#### Self-attention

**Fig. 11:**
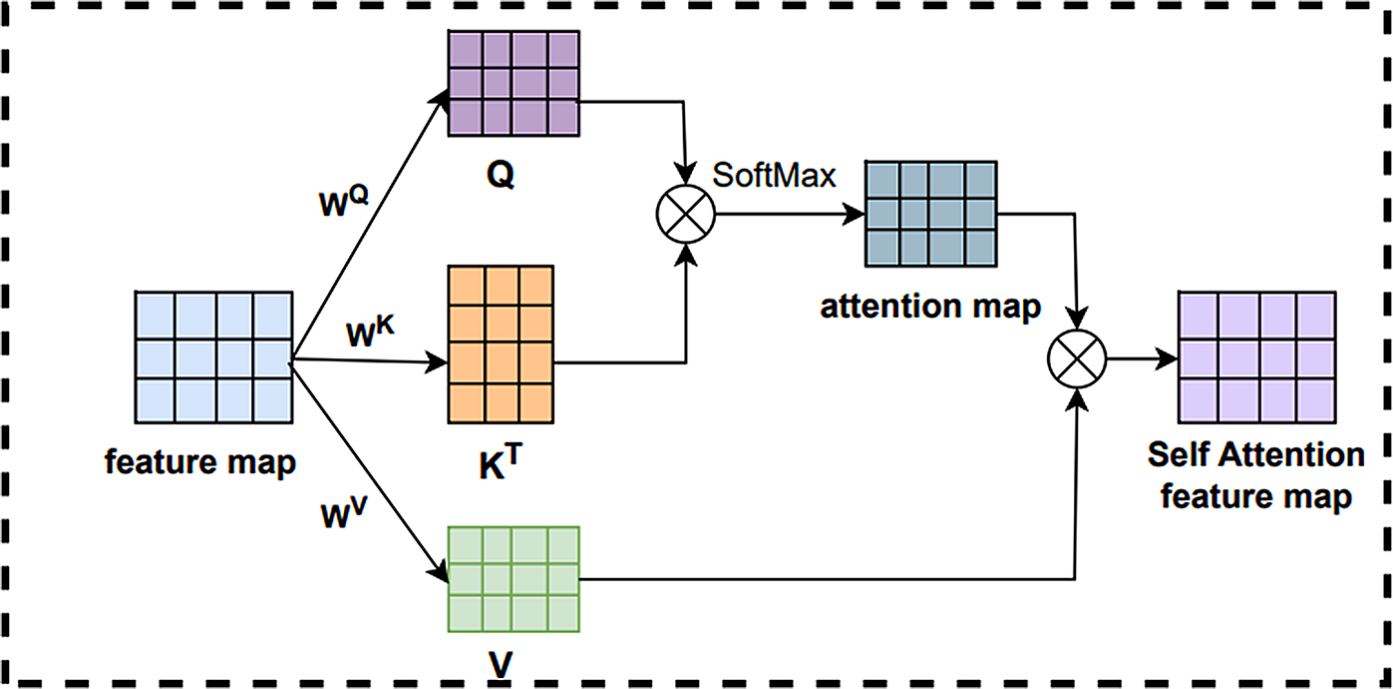
The structure of self-attention mechanisms

The Self-Attention mechanism automatically learns correlations between different positions in the input sequence, and it can handle longer and more complex input sequences. The Self-Attention mechanism can capture the global features of the input sequence while at the same time and focuses on the focal feature information. The structure of the Self-Attention mechanism is shown in Fig. 11.

First, the Self-Attention mechanism obtains the query vector Q, the key vector K, and the value vector V by linearly transforming of the input features. The dot product of the query vector Q with each key vector K yields a different similarity. Next, the similarity is SoftMax normalized to obtain the attention map. These attention maps quantify the importance of each element in the context. Finally, the attention weights and the value vector V are multiplied and summed to obtain the final Self-Attention representation.

#### Class weight

In bioinformatics, we often encounter the problem of sample imbalance. To solve this problem, there is a very useful parameter called class weight [42] during the training of deep learning models, which is used to automatically adjust the weights of unbalanced samples to balance the impact of unbalanced samples in training. In this study, the ratio of positive to negative samples in the baseline data set was 1:7.6. Therefore, we set the class weight parameter to 7.6 for positive sample weights and the class weight parameter to 1 for negative sample weights. With this setup, we boost the importance of the positive samples in the backpropagation process of the model, making the model more focused on learning the features of the positive samples.

### Performance evaluation

We used scientific evaluation metrics to comprehensively assess the performance of the i5mC-DCGA model, which included sensitivity (Sn), specificity (Sp), accuracy (Acc), and matthews correlation coefficient (MCC) [43]. These evaluation metrics can comprehensively reflect the performance of the model and help guide the optimization and improvement of the model. The formula for calculating the assessment indicators is specified in Eq. (3).

3 $$ \begin{array}{c}\left\{\begin{array}{c} Sp=\frac{TN}{TN+FP}\\ Sn=\frac{TP}{TP+FN}\\ Acc=\frac{TP+TN}{TP+TN+FP+FN}\\ MCC=\frac{TP\times TN-FP\times FN}{\sqrt{\left(TP+FP\right)\times \left(TP+FN\right)\times \left(TN+FP\right)\times \left(TN+FN\right)}}\end{array} \right.\end{array}$$

The above evaluation metrics were calculated based on the four metrics of True Positive (TP), True Negative (TN), False Positive (FP) and False Negative (FN) in the confusion matrix [44]. These metrics represent the accuracy and recall of the model in classifying samples, respectively. To characterize the performance of the model more accurately, we also introduced the Receiver Operating Characteristic Curve (ROC) and Area Under the Curve (AUC) metrics [45] for evaluation. The higher the values of all these evaluation metrics, the better the performance of the model.

In this study, to facilitate comparisons with other predictive models, we used five-fold cross-validation to train and validate the performance of the model, and the results of each one-fold cross-validation can be regarded as the results of an independent test. Finally, the evaluation metrics of the five-fold cross-validation are mean valued to ensure the reliability of the model.

## Data Availability

The data set and source code used in this study can be easily derived from https://github.com/leirufeng/i5mC-DCGA.

## Abbreviations

5mC: 5-Methylcytosine
CBAM: Convolutional Block Attention Module
DenseNet: Dense Convolutional Network
BiGRU: Bidirectional Gated Recurrent Unit
DNMTs: DNA methyltransferases
6mA: Adenine N-6 methylation
4mC: Cytosine N-4 methylation
m7G: Guanine N-7 methylation
5mC: Cytosine C-5 methylation
CNN: Convolutional Neural Network
ResNet: Residual Neural Network
RNN: Recurrent Neural Network
LSTM: Long Short-Term Memory Network
GRU: Gated Recurrent Unit
CCLE: Cancer Cell Line Encyclopedia
Sn: sensitivity
Sp: specificity
Acc: accuracy
MCC: matthews correlation coefficient
TP: True Positive
TN: True Negative
FP: False Positive
FN: False Negative
ROC: Receiver Operating Characteristic Curve
AUC: Area Under the Curve
